# Entwicklung des klinischen Risikomanagements in deutschen Krankenhäusern

**DOI:** 10.1007/s00103-022-03491-5

**Published:** 2022-02-08

**Authors:** Hans-Jürgen Bartz

**Affiliations:** Geschäftsbereich Qualitätsmanagement und klinisches Prozessmanagement, Universitätsklinikum Hamburg-Eppendorf, Martinistr. 52, 20246 Hamburg, Deutschland

**Keywords:** Digitalisierung, Qualitätsmanagement, Patientensicherheit, Peer-Review, Morbiditäts- und Mortalitätskonferenz, Digitization, Quality management, Patient safety, Peer review, Morbidity and mortality conference

## Abstract

Das klinische Risikomanagement (kRM) unterstützt Mitarbeitende im Gesundheitswesen dabei, Risiken bei der Patient:innenversorgung zu erkennen, zu reduzieren und zu bewältigen. Es ist für alle ambulanten und stationären Einrichtungen des deutschen Gesundheitswesens verpflichtend. Die Inhalte des kRM sind im Fünften Sozialgesetzbuch (SGB V), der Richtlinie des Gemeinsamen Bundesausschusses (G-BA), dem Patientenrechtegesetz und in Normen geregelt. Darüber hinaus gibt es die Empfehlungen des Aktionsbündnisses Patientensicherheit (APS). Der G‑BA weist explizit auf Mindeststandards hin. Diese gelten für das Risikomanagement, das Fehlermanagement, die Fehlermeldesysteme, das Beschwerdemanagement sowie die Nutzung von Checklisten bei operativen Eingriffen.

Der Gesetzgeber fordert, die Wirksamkeit des kRM regelmäßig zu überprüfen. Fragebogenerhebungen zum kRM in Deutschland zeigen insgesamt eine positive Entwicklung. Die Daten sind jedoch für eine umfassende Bewertung nicht hinreichend. Es sollten deshalb methodisch belastbare Verfahren entwickelt werden, die den Status des kRM deutlich häufiger und regelmäßiger überprüfen. Struktur‑, Prozess- und Ergebnisqualitätsdaten sollten systematisch erhoben und, bezogen auf die Einrichtungen, vergleichend dargestellt werden.

Chancen für das klinische Risikomanagement ergeben sich aus dem Global Action Safety Plan der Weltgesundheitsorganisation, Fortschritten in der Digitalisierung, der Integration des klinischen Risikomanagements in ein Organisationsrisikomanagement und der Verbesserung der Strukturqualität. Dem kRM muss in der täglichen Routine von Ärzt:innen und Pflegekräften noch mehr Raum gegeben werden. Dazu bedarf es Kompetenz und personeller Ressourcen im kRM. Diese sind in deutschen Krankenhäusern nicht ausreichend vorhanden.

**Zusatzmaterial online:**

Zusätzliche Informationen sind in der Online-Version dieses Artikels (10.1007/s00103-022-03491-5) enthalten.

## Abstract (en)

Clinical risk management supports healthcare workers in recognizing, reducing, and managing risks in patient care. It is mandatory for all outpatient and inpatient facilities in the German healthcare system. The contents of the clinical risk management are regulated in the Social Code (Title 5), the guidelines of the Federal Joint Committee, the Patients’ Rights Act, and the norms and recommendations of the Patient Safety Alliance. The Federal Joint Committee explicitly points out that minimum standards of risk management, error management, error reporting systems, complaint management in hospitals, and the use of checklists for surgical interventions must be implemented.

The legislator requires that the effectiveness of the clinical risk management be checked regularly. Questionnaire surveys on clinical risk management in Germany show an overall positive development. However, the data are not sufficient for a comprehensive assessment. Methodologically reliable procedures should therefore be developed that check the status of the clinical risk management much more frequently and regularly. The data measuring structure, process, and outcome should be collected systematically and presented in a comparative manner in relation to the facilities.

Opportunities for clinical risk management arise from the World Health Organization’s Global Action Safety Plan, advances in digitization, the integration of clinical risk management into organizational risk management, and the improvement of structural quality. Clinical risk management must be given even more space in the daily routine of doctors and nurses. This requires competence and human resources in this area. These are not sufficiently available in German hospitals.

## Einleitung

Auch bei der Organisation von Krankenhäusern können Pathologien auftreten, die das System anfällig für Fehler machen. Das „Syndrom des verletzlichen Systems“ (Vulnerable System Syndrome – VSS) hat 3 Leitsymptome [1]: Erstens neigen Organisationen mit einem VSS dazu, Einzelpersonen für Fehler verantwortlich zu machen (Blame Culture). Zweitens halten sie systemische Fehlerursachen bei der Fehleranalyse für sekundär. Drittens lassen sich diese Organisationen sehr stark von finanziellen Anreizen leiten. Ein wirksames klinisches Risikomanagement (kRM) kann dabei helfen, ein VSS zu verhindern, oder mindestens dafür sorgen, dass die Symptome beherrscht werden.

Das kRM umfasst nach Briner et al. [2] in Anlehnung an Middendorf [3] „die Strukturen, Prozesse, Instrumente und Aktivitäten, welche die Mitarbeitenden eines Krankenhauses unterstützen, die … Risiken bei der Patient:innenversorgung zu erkennen, zu reduzieren und zu bewältigen“. Das kRM sollte dazu in ein Qualitätsmanagementsystem (QMS) integriert sein, um mit dem PDCA-Zyklus (Plan Do Check Act, dt.: Planen-Ausführen-Überprüfen-Handeln) des QMS Verbesserungsprojekte steuern zu können. Das Aktionsbündnis Patientensicherheit (APS) hat eine Empfehlung formuliert, in der die Voraussetzungen für ein wirksames kRM sehr praktisch und umfassend beschrieben werden [4]. Die Identifikation der Risiken durch funktionierende Berichts- und Lernsysteme spielt dabei eine entscheidende Rolle. Hinzu kommt eine systematische Aufarbeitung mit definierten Protokollen, die eine systemische Fehleranalyse ermöglichen.

In diesem Artikel werden zunächst die Regelwerke erläutert, die für das kRM relevant sind. Dann wird der aktuelle Stand des kRM in Deutschland beschrieben. Dazu werden die Ergebnisse groß angelegter Befragungen vorgestellt. Die Inhalte des Artikels gelten, wie es auch in der Richtlinie des Gemeinsamen Bundesausschusses (G-BA) über grundsätzliche Anforderungen an ein einrichtungsinternes Qualitätsmanagement formuliert ist, für den ambulanten und stationären Sektor [5]. Der Schwerpunkt liegt in diesem Beitrag jedoch auf der stationären Patient:innenversorgung. Es wird auf die Notwendigkeit der Erhebungen des Istzustandes, der Implementierung und der systematischen Evaluation des Nutzens hingewiesen. Ziel der Übersicht ist es, den Status des kRM in deutschen Krankenhäusern zu beschreiben und Perspektiven aufzuzeigen.

## Regelwerke

Bei Regelwerken kann grundsätzlich zwischen *Gesetzen, Richtlinien, Leitlinien *und* Empfehlungen* sowie *Normen* unterschieden werden. *Gesetze* werden vom parlamentarischen Gesetzgeber beschlossen [6]. Das Patientenrechtegesetz vom 20.02.2013 verpflichtet Krankenhäuser, Risikomanagement- und Fehlermeldesysteme vorzuhalten. Es bündelt erstmals deren Rechte in einem eigenen Abschnitt des Bürgerlichen Gesetzbuchs (BGB; [7]). *Richtlinien* basieren auf einer gesetzlichen Grundlage und stellen bindende Handlungsanweisungen dar [8]. Der G‑BA hat in einer Richtlinie die grundsätzlichen Anforderungen für die erfolgreiche Einführung und Umsetzung eines einrichtungsinternen Qualitätsmanagements basierend auf dem Fünften Buch Sozialgesetzbuch (SGB V) festgelegt [9]. In der Richtlinie wird explizit darauf hingewiesen, dass Mindeststandards des Risikomanagements eingehalten werden müssen. *Leitlinien* unterstützen mit einer systematischen Bewertung der Evidenz die Entscheidungsfindung [10]. *Empfehlungen* sind Ratschläge zu ausgewählten praxisrelevanten Fragestellungen [8]. Das Aktionsbündnis Patientensicherheit (APS) gibt Handlungsempfehlungen heraus, um die Patient:innensicherheit in deutschen Krankenhäusern zu verbessern. Die Handlungsempfehlung „Anforderungen an klinische Risikomanagementsysteme im Krankenhaus“ gibt den Rahmen für das kRM vor [11]. Für das kRM in Krankenhäusern stehen grundsätzlich 2 spezifische *Normen* zur Verfügung. Die DIN ISO 31000:2018 legt Leitlinien für das Risikomanagement fest [12]. Die Norm beinhaltet eine generische Methodik für das Management von Risiken verschiedenster Art. Die neue österreichische ÖNORM-Reihe D 4900 wurde im Januar 2021 publiziert. Das neue Regelwerk beschreibt die Anforderungen an ein Risikomanagement und hilft mit Leitlinien und Checklisten bei der praktischen Umsetzung der ISO 31000:2018 [13].

## Aktueller Stand des kRM in Deutschland

Aussagekräftige Übersichtsarbeiten zum aktuellen Stand des kRM in Deutschland sind rar. Die verwertbaren Studien werden in diesem Abschnitt zusammengefasst. Grundsätzlich finden sich Informationen zum Stand des kRM in Deutschland auch im Kapitel „Qualitätsmanagement“ der Qualitätsberichte der Krankenhäuser. Diese werden vom Institut für Qualitätssicherung und Transparenz im Gesundheitswesen (IQTIG) herausgegeben. Das Kapitel 3.13 des Qualitätsberichts enthält Angaben zum Umgang mit Risiken in der Patient:innenversorgung. Eine systematische Auswertung der Qualitätsberichte zum kRM findet beim IQTIG jedoch nicht statt, da eine Beauftragung durch den G‑BA fehlt. Dies teilte das IQTIG am 31.05.2021 auf Anfrage mit.

Das APS hat eine Übersicht der Instrumente des kRM in ihren Empfehlungen „Anforderungen an klinische Risikomanagementsysteme im Krankenhaus“ veröffentlicht, die in deutschen Krankenhäusern implementiert sind [4]. Über den Durchdringungsgrad der einzelnen Instrumente fehlen, mit Ausnahme der unten zitierten Quellen, genauere Informationen.

### Befragung zum Einführungsstand des kRM in Deutschland (2010)

Die erste systematische Untersuchung zum klinischen Risikomanagement in Deutschland wurde im Jahr 2010 durchgeführt [14]. Es wurden 1815 Krankenhäuser angeschrieben. Die Rücklaufquote betrug 27 % (*n* = 484). Die Autor:innen fassen ihre Ergebnisse so zusammen:

- Das kRM war selten systematisch in die Unternehmensstrategie integriert.
- Die Durchdringung und Implementierung von kRM-Zyklen war verbesserungswürdig.
- Umsetzungsstrategien zur Entwicklung der kRM-Organisation waren vorhanden.
- Es gab wenig Personal, das ausschließlich für das kRM eingesetzt wurde.
- Besprechungen zu kritischen Vorfällen waren vielfach unzureichend organisiert.
- Fast 2 Drittel der Befragten benötigten externe Beratung bei der Implementierung des kRM.
- Im kRM mangelte es an der Zusammenführung der Informationen.
- Fast 48 % der Befragten hatten bereits ein lokales CIRS (Critical Incident Reporting System, dt.: Berichtssystem über kritische Vorkommnisse) implementiert.
- Bei der Risikoanalyse waren standardisierte Vorgehensweisen die Ausnahme.
- Maßnahmen zur Verbesserung der Arzneimitteltherapiesicherheit, der Sturz- und Dekubitusprophylaxe, der Hygiene, der sicheren Patient:innenidentifikation und des OP- und Entlassungsmanagements waren überwiegend etabliert. Dies jedoch nicht flächendeckend.
- Die klinischen Risiken wurden überwiegend mit Routinedaten und eigenen Kennzahlen überwacht.
- Die externe Berichterstattung wurde sehr zurückhaltend gehandhabt. Dies ist ein Anhalt für die Unsicherheit im Umgang mit dem Thema Patient:innensicherheit im öffentlichen Raum.
- Die Krankenhäuser sahen Verbesserungsbedarf bei finanziellen und personellen Ressourcen, in der Organisations- und Fehlerkultur sowie in der internen Zusammenarbeit.
- Der Fortbildungsbedarf im Bereich des kRM war hoch.
- Die Risikoschwerpunkte waren unabhängig von der Größe der Krankenhäuser identisch: Schnittstellenprobleme 46,5 %, Arzneimitteltherapie 34,4 % und Krankenhaushygiene 32,2 %.
- 77,5 % der befragten Krankenhäuser schätzten Nutzen und Wirksamkeit ihrer kRM-Maßnahmen positiv ein. Größere Krankenhäuser waren in der kRM-Umsetzung weiter als kleinere.

### Befragung zum Einführungsstand des kRM in Deutschland (2015)

Die letzte systematische Bestandsaufnahme zum kRM in Deutschland wurde im Jahre 2015 im Auftrag des G‑BA durchgeführt [15]. Die Ergebnisse wurden darin mit der Studie von Lauterberg et al. [14] aus dem Jahre 2010 verglichen. Folgende Dimensionen wurden untersucht: Strategien und Ziele, Strukturen des kRM, Risikomanagementprozess. Es wurden 2617 Krankenhäuser und Rehabilitationskliniken angeschrieben. Die Rücklaufquote von 22 % (*n* = 572) entsprach der Verteilung der Grundgesamtheit.

Insgesamt zeigten sich deutliche Unterschiede im Umsetzungsgrad bei Strategie, Zielen und Strukturen. Optimierungspotenzial gab es bei der Integration von Analyseergebnissen aus den verschiedenen Informationsquellen des kRM. Es bestand noch großer Fortbildungsbedarf bei der Gestaltung des Risikomanagementprozesses. Die Krankenhäuser wünschten sich vor allem eine klare Regelung von Verantwortlichkeiten im kRM. Die Autor:innen hielten die Befragungsmethodik für ein geeignetes Überwachungsinstrument für die Evaluation des kRM in Deutschland. Mit den Ergebnissen könnten Strategieentscheidungen auf politischer Ebene vorbereitet werden. Die Ergebnisse der Befragung sind in vereinfachter Skalierung im Onlinematerial zu finden. Ferner ist eine Auswahl wichtiger Ergebnisse der Entwicklung des kRM von 2010 bis 2015 in Tab. 1 dargestellt.

**Table 1:** 

Die Anzahl der Krankenhäuser mit Zertifizierung war rückläufig
Es wurden häufiger strategische Ziele für das kRM formuliert
Die Verantwortlichkeiten im kRM waren häufiger klar definiert, dokumentiert und zugeordnet
Fortbildungsmaßnahmen wurden intensiviert
Das kRM war häufiger Thema bei Sitzungen der Krankenhausleitung
Die Transparenz für Patient:innen bei Fehlern und kritischen Ereignissen wurde verbessert
Beratung war vor allem noch bei der Einführung von CIRS und kRM-Prozess nötig
Fallkonferenzen waren mehr zur Routine geworden. Bei Morbiditäts- und Mortalitätskonferenzen gab es Fortschritte in der Systematik
Das Beschwerdemanagement wurde fast überall systematisch zur Risikoidentifikation genutzt
Die systematische Analyse von Kennzahlen war weitverbreitet
Die direkte Beobachtung klinischer Abläufe gehörte in fast allen Einrichtungen zur Routine
Die Auswertung von Patient:innenschadensfällen war in das kRM integriert
Mitarbeiter:innenbefragungen waren eher weniger verbreitet
CIRS war in fast allen Krankenhäusern implementiert
Das einrichtungsübergreifende CIRS wurde genutzt
Fast alle Krankenhäuser definierten kritische Ereignisse, die gemeldet werden sollen
Schulungen und zeitnahes Feedback zu Berichten hatten sich verbessert
Die Analyse von CIRS-Berichten inklusive Maßnahmenmanagement hatte sich verbessert
Fortschritte gab es bei: Patient:innenidentifikation, MRSA-Screening, Sturz-Assessment, Nutzung perioperativer Checklisten, Umsetzung eines systematischen Entlassungsmanagements
Das Simulatortraining wird im Vergleich zu anderen Instrumenten als weniger relevant betrachtet
Nosokomiale Infektionen wurden mit 93 % auch 2015 fast flächendeckend überwacht
Die Relevanz des Fortbildungsbedarfs zum kRM-Prozess wurde als gleich hoch bewertet
Die Schwerpunktthemen lagen immer noch an den Schnittstellen (z. B. Arzneimitteltherapie)
Das einrichtungsübergreifende CIRS war noch ohne Einfluss auf das Lernen in den Organisationen

*CIRS* Critical Incident Reporting System, *MRSA* methicillinresistenter Staphylococcus aureus

Die Autor:innen weisen auf Limitationen ihrer Studie hin. Dazu gehören ein Selektionsbias (Teilnahme von Einrichtungen mit bereits installiertem kRM), die Selbstauskunftsebene (fehlende Validierung der Antworten), fehlende Deckungsgleichheit der Stichproben der Jahre 2010 und 2015 und geänderte Skalenniveaus. Um die Entwicklungen im kRM zu evaluieren, fordern die Autor:innen ein systematisches nationales Monitoring des kRM, was Informationen für politische Entscheidungen liefert.

Im selben Jahr veröffentlichten Rothmund et al. die Ergebnisse einer Onlinebefragung von Chirurg:innen [16]. Diese nutzten sehr häufig Instrumente zur Fehlerprävention. Etwa 90 % wendeten in ihren Kliniken Instrumente wie Checklisten, Markierungen des Eingriffsortes und Infektionsstatistiken an. Etwa 75 % nutzten Instrumente wie CIRS, Identifikationsarmbänder und Morbiditäts- und Mortalitätskonferenzen. Präoperative Checklisten, Seitenmarkierung, Schulungen vor Arbeitsantritt und Geräteeinweisungen wurden von den Befragten als am wirksamsten eingeschätzt. CIRS wurde nur von der Hälfte der Befragten eine Wirksamkeit zugemessen. Rothmund et al. kamen zu dem Schluss, dass die Instrumente zur Verbesserung der Patient:innensicherheit in chirurgischen Kliniken vorhanden sind. An deren optimaler Anwendung in der täglichen Praxis muss jedoch noch gearbeitet werden.

### Evaluation der Handlungsempfehlungen des APS (2019)

Bisher hat das APS 24 Handlungsempfehlungen, 9 Patient:inneninformationen, 47 Stellungnahmen und 97 Pressemitteilungen publiziert (Stand 09/2021). Die Handlungsempfehlungen sollen helfen, Patient:innenschäden zu reduzieren. Voraussetzung dafür ist, dass diese von den Akteur:innen im Gesundheitswesen genutzt werden. Dies wurde in einer quantitativen Evaluationsstudie geprüft [17]. Qualitätsmanager:innen aus 769 deutschen Akutkliniken wurden in einer Onlinebefragung angeschrieben. 202 Kliniken nahmen an der Befragung teil (Rücklaufquote 26,3 %). 135 Fragebögen (17,6 %) wurden ausgewertet. 80,7 % (*n* = 109) der Kliniken kannten die Handlungsempfehlungen des APS. Davon nutzten 85,3 % (*n* = 93) diese auch in ihrer Klinik. Als Gründe dafür wurden die fachliche Expertise und die strukturierte Aufbereitung der Handlungsempfehlungen angegeben. Die Befragten schlugen zur Verbesserung Umsetzungsberichte und Praxisbeispiele vor. Weitere Handlungsempfehlungen sollten die Themen Patient:innenidentifikation und Hygiene behandeln.

Die Studie kam zu dem Schluss, dass die Handlungsempfehlungen einen relevanten Beitrag zur Verbesserung der Patient:innensicherheit leisten. Immerhin 19,3 % der Befragten kannten die Handlungsempfehlungen nicht. Deshalb sollten, nach Meinung der Autor:innen, Methoden entwickelt werden, die zu einer besseren Distribution und Integration der Handlungsempfehlungen in den Klinikalltag beitragen.

### Evaluation von Peer-Review-Verfahren in Deutschland (2015)

Das Peer-Review gehört zu den Instrumenten des Risikomanagements, die auch vom APS empfohlen werden. Darunter versteht man eine Überprüfung (Review) und Bewertung durch Gleichgestellte (Peers). Die Bundesärztekammer hat das Curriculum „Ärztliches Peer-Review“ entwickelt [18]. Ziel des Peer-Reviews ist es, Gesundheitseinrichtungen bei ihren Bemühungen um nachhaltige Verbesserung von Qualität und Sicherheit zu unterstützen. Die Bundesärztekammer gab auf Nachfrage an, dass von 2010 bis April 2019 1820 Peers nach dem oben genannten Curriculum durch die Landesärztekammern geschult wurden.

Die Initiative Qualitätsmedizin (IQM) hat zusammen mit deutschen Krankenhäusern ein Peer-Review-Verfahren installiert [19]. Die ersten freiwilligen Reviews fanden 2010 statt. Eberlein-Gonska et al. lieferten 2015 eine gründliche Bestandsaufnahme des Peer-Review-Verfahrens der IQM [20]. Die Autor:innen sichteten in einer systematischen und deskriptiven Analyse alle Protokolle von Peer-Reviews, die im Zeitraum 2010 bis 2014 durchgeführt wurden. Ziel der Studie war es, die Leistungsfähigkeit des Peer-Review-Verfahrens im zeitlichen Verlauf darzustellen. 294 Peer-Reviews wurden in der Untersuchung berücksichtigt. Die Stichprobe bildete die Grundgesamtheit der deutschen Krankenhauslandschaft sehr gut ab. Wichtige Ergebnisse und Schlussfolgerungen der Bestandsaufnahme waren: Das Verfahren hat sich gut etabliert. Das externe Peer-Team schätzte den Verbesserungsbedarf generell höher ein als die Ärzt:innen des Krankenhauses. Es konnten relevante medizinische Auffälligkeiten identifiziert werden. Diagnostik und Behandlung wurden im Verlauf adäquater und zeitgerechter.

Die IQM publizierte von 2010 bis 2012 ein Jahrbuch. Seit 2014 gibt es das Handbuch IQM. Die aktuelle Ausgabe ist die 2. Auflage des Handbuchs aus dem Jahr 2017. Derzeit laufen die Planungen für eine Neuausgabe, die voraussichtlich 2022 erscheinen wird. Damit können auch Krankenhäuser, die nicht Mitglied bei IQM sind, die Ergebnisse nutzen. So können sie die eigene Patient:innenversorgung sehr gut reflektieren.

Darüber hinaus existieren spezielle Peer-Reviews in der Intensivmedizin [21, 22]. Diese Verfahren haben hat sich seit 2006 in Deutschland aus Netzwerken von Intensivmediziner:innen entwickelt. Es gab mehrere Jahre Pilotprojekte von intensivmedizinischen Fachgesellschaften. Wichtigster Bestandteil der Peer-Reviews sind die 10 evidenzbasierten intensivmedizinischen Qualitätsindikatoren (Tab. 2; [23]). Ziel des Peer-Reviews ist es, evidenzbasiertes Wissen fachübergreifend und multiprofessionell in der täglichen Praxis auf den Intensivstationen etablieren zu helfen. Dafür wurde ein Fragebogen mit 60 Fragen mit den Dimensionen Struktur‑, Prozess- und Ergebnisqualität entwickelt. Innerhalb der Dimensionen gab es Fragen zur Organisation der Intensivstation, zum Personal und zu den Patient:innen. Es haben von 2010 bis 2021 über 200 Peer-Reviews nach dem Verfahren der Deutschen Interdisziplinären Vereinigung für Intensiv- und Notfallmedizin (DIVI) stattgefunden. Grundsätzlich zeichnet sich das Verfahren durch eine hohe Akzeptanz aus und führt auf den besuchten Stationen zu positiven Veränderungen. Eine zusammenfassende Übersicht der Befunde existiert nicht. Seit geraumer Zeit finden Peer-Reviews nur noch vereinzelt statt. Die Methode ist sehr hilfreich, aber gleichzeitig auch ressourcenintensiv für beide Seiten. Künftig sollen die Peer-Reviews von der DIVI wieder stärker beworben werden.

**Table 2:** 

Nr.	Hauptindikatoren
I	Tägliche multiprofessionelle und interdisziplinäre Visite mit Dokumentation von Tageszielen
II	Management von Sedierung, Analgesie und Delir
III	Patient:innenadaptierte Beatmung
IV	Frühzeitige Entwöhnung von einer invasiven Beatmung (Weaning)
V	Überwachung der Maßnahmen zur Infektionsprävention
VI	Maßnahmen zum Infektionsmanagement
VII	Frühe enterale Ernährung
VIII	Dokumentation einer strukturierten Patient:innen- und Angehörigenkommunikation
IX	Frühmobilisation
X	Leitung der Intensivstation

### Befragungen zu Morbiditäts- und Mortalitätskonferenzen

In Morbiditäts- und Mortalitätskonferenzen (MMK) werden retrospektiv Komplikationen, ungewöhnliche Behandlungsverläufe und unerwartete Todesfälle aufgearbeitet. Ziel ist es, daraus zu lernen und Wiederholungen zu vermeiden. Kognitive und systemische Faktoren stehen bei der MMK im Vordergrund. Dadurch unterscheidet sich diese von klinischen Fallkonferenzen, Tumorboards oder Transplantationskonferenzen [24]. Die MMK soll das individuelle und organisationale Lernen in Krankenhäusern unterstützen. Um die MMK wirksam zu gestalten, ist Methodenkompetenz notwendig. Diese bezieht sich vor allem auf Zwischenfallanalyse, Moderation und Konfliktmanagement. Die Kompetenzen müssen in externen und internen Schulungen erworben werden. Diese werden von medizinischen Fachgesellschaften und Verbänden angeboten [25].

Zum Stand der Morbiditäts- und Mortalitätskonferenzen (MMK) haben Schwappach et al. eine Übersicht geliefert [26]. Dies ist die einzige aktuelle Erhebung, die für Deutschland verfügbar ist. Sie bezieht sich auf das Bundesland Niedersachsen. Ziel der Querschnittstudie war es, in einer Onlinebefragung den Iststatus und Verbesserungspotenziale bei den MMK zu erfassen. Die Rücklaufquote lag bei 50 %. Auffällig war, dass in den MMK zunehmend Probleme bei Prozessen (92 %) und Zusammenarbeit (64 %) thematisiert wurden. 37 % der Befragten gaben an, fachliche Themen zu diskutieren. Struktur und Prozess der MMK in Niedersachsen sind sehr heterogen. Die Zufriedenheit der befragten Ärzt:innen, die MMKs organisieren und durchführen, ist hoch (85 %). Die Wirksamkeit wird subjektiv ebenfalls hoch eingeschätzt (93 %). Dennoch sehen die meisten der Befragten Verbesserungspotenzial (58 %). Die Autor:innen folgern, dass die hohe Akzeptanz und die Motivation sehr gute Voraussetzungen dafür sind, die MMK in der klinischen Versorgung noch besser zu etablieren.

### Befragungen zum Beschwerdemanagement

Wie oben beschrieben, ist das Beschwerdemanagement ein Baustein des kRM. Laut einer Befragung von Beschwerdemanager:innen in deutschen Krankenhäusern durch den Bundesverband Beschwerdemanagement für Gesundheitseinrichtungen e. V. betreffen 20,4 % der Beschwerden in Kliniken die Organisation und Logistik, 18,5 % die Kommunikation und 15,5 % die Ausstattung der Klinik [27]. 10,8 % der Beschwerden werden als hoch relevant bezeichnet (Verdacht auf Behandlungsfehler, Verletzung der Persönlichkeitsrechte, Gefährdung der Sicherheit). Nur 38,0 % der Befragten verfügten über eine Beschwerdemanagementsoftware. Dies erklärt möglicherweise, dass für die Bearbeitung von 1000 Beschwerden durchschnittlich 1,9 Vollkräfte benötigt werden. Bei 70,6 % der Befragten war das Beschwerdemanagement Teil des Qualitätsmanagements. 79 % der Befragten entwickelten ihre Maßnahmen zusammen mit dem Risikomanagement des Krankenhauses. Dies spricht für eine gute Integration des Beschwerdemanagements in das kRM. In der Untersuchung, die bereits zum 7. Mal stattfand, wurden 2012 Personen angeschrieben. 280 Personen (13,9 %) gaben eine Rückmeldung für insgesamt 343 Klinikverbünde und 468 Standorte.

## Perspektiven

### Neue Befragungen

Das Bundesministerium für Gesundheit hat eine neue bundesweite Befragung zum Umsetzungsstand des klinischen Risikomanagements in Krankenhäusern und Rehabilitationskliniken in Deutschland unter der Projektleitung des APS bewilligt. Die Befragung wird vom Institut für Patientensicherheit, dem Deutschen Krankenhausinstitut und der Hochschule RheinMain konzipiert und durchgeführt. Die Befragung baut auf den 2010 und 2015 erhobenen Daten auf. Weiterführende Fragen sollen ergänzt werden. Die Projektlaufzeit beträgt ein Jahr (01.08.2021 bis 31.07.2022).

Die Befragungsinitiativen sind sehr begrüßenswert. Die Selbstauskunftsebene reicht jedoch nicht aus, um die Funktionsfähigkeit eines Systems umfassend und zuverlässig zu beurteilen. Darüber hinaus bleiben die Befragungsergebnisse ohne zwingende Konsequenz, da Ziele nicht definiert sind und Verbesserungsmaßnahmen nicht abgeleitet werden müssen. Es wäre deshalb sinnvoll, adäquate Indikatoren einzuführen, die die Leistungsfähigkeit des Systems tatsächlich messen und Trendbeurteilungen ermöglichen. Damit könnten Veränderungen deutlich gemacht werden. Klar definierte und validierte Patient:innensicherheitsindikatoren, die regelmäßig berichtet werden müssen, wären hierzu sehr geeignet.

### Global Safety Action Plan der Weltgesundheitsorganisation

Die Weltgesundheitsorganisation (WHO) will mit ihrem globalen Aktionsplan von 2021 die Quellen vermeidbarer Risiken und Schäden für Patient:innen und Angestellte im Gesundheitssektor bis 2030 eliminieren [28]. Dies soll durch die Entwicklung von Strategien und Maßnahmen geschehen, die auf Wissenschaft, Patient:innenerfahrung, Systemdesign und Partnerschaften basieren. Die WHO stellt in ihrem Aktionsplan 7 strategische Ziele vor (Tab. 3). Darüber hinaus werden in dem Aktionsplan ausführlich Messverfahren und Berichtssysteme beschrieben, mit denen die Wirksamkeit der Maßnahmen gemessen werden kann. Die WHO präsentiert darin 7 sogenannte Kernindikatoren, die insgesamt 47 erweiterte Indikatoren enthalten. Beide Indikatorentypen können sehr gut als Rahmen für ein Monitoring des kRM in Deutschland genutzt werden.

**Table 3:** 

Nr.	Strategische Ziele
1	Die Eliminierung aller vermeidbaren Patient:innenschäden muss überall zu einer Geisteshaltung und zu einem Grundsatz für die Planung und Durchführung der Gesundheitsversorgung werden
2	Aufbau hochzuverlässiger Gesundheitssysteme und -organisationen, die Patient:innen täglich vor Schaden bewahren
3	Gewährleistung der Sicherheit aller klinischen Prozesse
4	Einbindung und Befähigung von Patient:innen und deren Familien, um den Weg zu einer sichereren Gesundheitsversorgung zu erleichtern und zu unterstützen
5	Inspiration, Ausbildung, Qualifizierung und Schutz aller Beschäftigten des Gesundheitswesens, damit diese zur Gestaltung und Umsetzung sicherer Versorgungssysteme beitragen können
6	Gewährleistung eines ständigen Informations- und Wissensflusses, um die Risikominderung, die Verringerung vermeidbarer Schäden und die Verbesserung der Sicherheit in der Versorgung zu fördern
7	Entwicklung und Aufrechterhaltung von sektorübergreifenden und multinationalen Synergien, Partnerschaften und Solidarität zur Verbesserung der Patient:innensicherheit und der Qualität der Versorgung

### Digitalisierung

Der Fortschritt in den Informationstechnologien folgt einem disruptiven Muster. Es lässt sich deshalb nicht sicher vorhersagen, was künftig technisch möglich sein wird. In diesem Abschnitt soll auf das elektronische klinische Arbeitsplatzsystem (KAS; englisch: Electronic Health Record, EHR) eingegangen werden. Das KAS ist das zentrale Werkzeug im klinischen Alltag von Ärzt:innen und Pflegekräften [29]. Es verdient deshalb besondere Berücksichtigung. Es werden jetzt, ohne Anspruch auf Vollständigkeit, Instrumente genannt, die im KAS für das kRM genutzt werden können:

#### Natural Language Processing (NLP).

Darunter versteht man jeden computergestützten Algorithmus, der natürliche Sprache verarbeitet, erweitert und transformiert, sodass dieser für Berechnungen genutzt werden kann [30]. Idealerweise liegen die Daten im KAS in strukturierter Form vor. Das NLP verwandelt unstrukturierte Daten in strukturiere und damit auswertbare Daten.

#### Decision-Support-Systeme (DSS).

Dies sind Systeme, die den Behandlern schnell und zuverlässig relevante Informationen für Entscheidungen zur Verfügung stellen (z. B. personalisierte Dashboards).

#### Global-Trigger-Tool (GTT).

Ein GTT erlaubt die Detektion von Schwachstellen im System durch das Scannen medizinischer Information (Laborergebnisse, Bildgebung etc.; [31]). In diesen Kontext gehören auch medizinische Scores, um Risikopatient:innen schnell identifizieren zu können. So können der Medical Early Warning Score (MEWS) oder alle relevanten Scores aus der Intensivmedizin automatisiert aus dem KAS erhoben und ausgewertet werden [32]. Ein KAS ermöglicht damit automatische Rückmeldungen von Behandlungsinformationen an die Behandler vor Ort.

#### Closed Loop Medication Management (CLMM).

Hierunter versteht man einen vollautomatisierten digitalen Medikationsprozess, von der Verschreibung bis zur Verpackung in der Klinikapotheke mittels Robotertechnologie. Die Arzneimitteltherapiesicherheit (AMTS) wird dadurch deutlich verbessert [33].

#### Patient Reported Outcomes Measures (PROMs).

Hiermit ist die Bewertung der Behandlungsqualität durch systematische Nachbefragungen von Patient:innen gemeint. PROMs können in ein modernes KAS integriert werden. Künftig kann damit auch das Potenzial mobiler Technologien, die von Patient:innen genutzt werden (Wearables), voll ausgeschöpft werden [34]. Standardsets für PROMs stellt das International Consortium for Health Outcomes Measurement (ICHOM) zur Verfügung [35].

#### Klinische Pfade.

Diese standardisieren die Abläufe entlang einer Zeitachse. Dadurch sind Prozessanalysen einfacher möglich. Ein KAS kann die prozessorientierte Darstellung von Behandlungsabläufen unterstützen. Wenn Wissen in Form von Leitlinien und Standard Operating Procedures (SOPs) direkt ins KAS integriert und regelmäßig aktualisiert wird, profitiert davon das klinische Risikomanagement. Standard Operating Procedures (SOPs) berücksichtigen, im Gegensatz zu Leitlinien und anderen Regeln, die Besonderheiten des Krankenhauses.

### Organisationsrisikomanagement

Im überwiegenden Teil der deutschen Krankenhäuser geschieht das Risikomanagement in Organisationseinheiten, die mehr oder weniger gut vernetzt sind. Sogenannte Silostrukturen sind in deutschen Kliniken keine Seltenheit. Ein Organisationsrisikomanagement (synonym: integriertes Risikomanagement) gibt der Unternehmensführung, als sinnvolle Erweiterung schon bestehender Steuerungsinstrumente, einen geordneten Überblick über die Risiken, denen die Organisation ausgesetzt ist. Betriebliche, klinische, informationstechnologische und Compliancerisiken können zusammen betrachtet werden.

Das kRM sollte gleichberechtigt mit den anderen Risikobereichen in ein Organisationsrisikomanagement integriert sein. Wenn sich klinische Risiken realisieren, hat dies, wie bei anderen Risiken auch, finanzielle Auswirkungen. Nach Analysen der Organisation für wirtschaftliche Zusammenarbeit und Entwicklung (OECD) können 15 % der Kosten im Gesundheitswesen auf Sicherheitsmängel in der Patient:innenversorgung zurückgeführt werden [36]. Die OECD nennt 5 Themenfelder, die maßgeblich zu diesen Kosten beitragen: nosokomiale Infektionen, venöse Thrombembolien, Druckgeschwüre, Medikationsfehler und Diagnosefehler. Um diese Themen sollte sich das kRM der Krankenhäuser deshalb vordringlich kümmern. Organisationen, die dem kRM eine hohe Bedeutung beimessen, handeln am Ende wirtschaftlicher als jene, die dies nicht tun [37]. Themen des klinischen Risikomanagements gehören, nicht nur deshalb, fest auf die Agenda von Leitungssitzungen.

### Verbesserung der Strukturqualität

Das kRM braucht eine gute Strukturqualität mit ausreichend kompetentem Personal und Sachmitteln. Dies ist erste Voraussetzung für gute Analysen, Maßnahmenpläne und deren Umsetzung. Die Personalkapazitäten für das kRM sind schwer zu bestimmen und deshalb nicht explizit festgelegt. Informelle Recherchen und Gespräche im Qualitätsausschuss des Verbands der Universitätsklinika Deutschlands (VUD) geben jedoch Anlass zu der Annahme, dass es an Personalressourcen im kRM mangelt. Dies bezieht sich sowohl auf den ärztlichen und pflegerischen als auch auf den administrativen Bereich. In der oben zitierten Befragung sahen immerhin 73 % der Befragten einen Verbesserungsbedarf bei personellen Ressourcen [38]. Auch Gausmann weist auf die Knappheit der Ressourcen hin [39]. In Krankenhäusern seien nicht nur deutlich geringere personelle Ressourcen für das Sicherheitsmanagement als in anderen Industrien vorhanden, es sei auch das Fehlen von Weisungsbefugnissen festzustellen. Er führt dies auf eine noch zu geringe Würdigung des klinischen Risikomanagements in der Krankenhauswelt zurück.

Der überwiegende Teil der Arbeit im kRM wird, wie andere Aufgaben im Qualitätsmanagement, in der Regel zusätzlich zur normalen Arbeit („on top“) erledigt. Dies sorgt für Schwierigkeiten bei der Implementierung und Entwicklung. In der Implementierungswissenschaft ist das COM-B-Modell eine anerkannte Methodik [40]. Das Akronym steht für Capability (Fähigkeit), Opportunity (Gelegenheit), Motivation (Motivation), Behaviour (Verhalten). *Fähigkeit*: Die Akteure müssen den Risikomanagementkreislauf anwenden können. Dies gilt vor allem für die systemische Zwischenfallanalyse (Error and Risk Analysis, ERA; London-Protokoll). Um Personen mit Fähigkeiten auszustatten, sind Schulungen notwendig. Diese brauchen Zeit. Das gilt ebenso für Analysen in MMKs und Fallkonferenzen. *Gelegenheit*: Es muss Zeit vorhanden sein, die Fähigkeiten anzuwenden. *Motivation*: Es müssen Anreize geschaffen werden. Die Arbeit muss sich lohnen. Um Motivation zu erzeugen, muss die Arbeit im kRM zu verwertbaren Ergebnissen führen. *Verhalten*: Die 3 oben genannten Faktoren bewirken, dass eine Verhaltensänderung (Arbeit im kRM) erreicht wird.

Für ein wirksames kRM sollten Krankenhäuser ein zentrales kRM einrichten (Top-down-Ansatz). Noch wichtiger ist es jedoch, Ärzt:innen und Pflegekräften feste Zeitkontingente für das kRM zur Verfügung zu stellen (Bottom-up-Ansatz). Diese Berufsgruppen sind es, die am „sharp end of patient care“, wie es Cross et al. [41] kürzlich noch einmal formuliert haben, klinisches Risikomanagement betreiben. Sie müssen Fähigkeiten, Gelegenheit und Motivation haben, den Risikomanagementprozess in ihren Krankenhäusern umzusetzen. Dann kann das klinische Risikomanagement in den Krankenhäusern nachhaltig verbessert werden.

## Fazit

Das klinische Risikomanagement (kRM) ist Pflicht für ambulante und stationäre Einrichtungen des deutschen Gesundheitswesens. Die Inhalte und Methoden des kRM sind im Sozialgesetzbuch V (SGB V), der Richtlinie des Gemeinsamen Bundesausschusses (G‑BA), dem Patientenrechtegesetz und in Normen geregelt. Darüber hinaus gibt es eine Reihe von Empfehlungen des Aktionsbündnisses Patientensicherheit (APS). Der Gesetzgeber fordert, die Wirksamkeit des kRM regelmäßig zu überprüfen. In der Literatur finden sich einige Statusberichte zum kRM in Deutschland. Die Ergebnisse aus den Fragebogenerhebungen zeigen insgesamt eine positive Entwicklung. Die Daten reichen jedoch für eine hinreichende Bewertung nicht aus. In Ergänzung zu diesen Erhebungen sollten deshalb methodisch belastbare Verfahren entwickelt werden, die den Status des kRM deutlich öfter und vor allem regelmäßig überprüfen. Die Struktur‑, Prozess- und Ergebnisqualitätsdaten sollten, bezogen auf die Einrichtungen, vergleichend dargestellt werden. Chancen für das kRM ergeben sich aus dem globalen Aktionsplan der Weltgesundheitsorganisation (WHO), Fortschritten in der Digitalisierung, der Einführung eines Organisationsrisikomanagements sowie der Verbesserung der Strukturqualität. Dem kRM muss in der täglichen Routine von Ärzt:innen und Pflegekräften noch mehr Raum gegeben werden. Dazu bedarf es Kompetenz im kRM sowie personeller Ressourcen. Diese sind noch nicht überall ausreichend vorhanden.

## Supplementary Information




