# Supplementary material for: Development of clinical risk management in German hospitals
Source: Bundesgesundheitsblatt Gesundheitsforschung Gesundheitsschutz. 2022 Feb 8;65(3):293–301. [Article in German] doi: 10.1007/s00103-022-03491-5 (PMC8888368; doi:10.1007/s00103-022-03491-5)
Supplement: Supplementary file 1 [file 103_2022_3491_MOESM1_ESM.pdf]

Bundesgesundheitsblatt – Gesundheitsforschung – Gesundheitsschutz, Ausgabe 3/2022

Onlinematerial zum Beitrag:

Entwicklung des klinischen Risikomanagements in deutschen Krankenhäusern

Hans-Jürgen Bartz[1]

[1] Geschäftsbereich Qualitätsmanagement und klinisches Prozessmanagement am Universitätsklinikum Hamburg-Eppendorf, Hamburg, Deutschland

Korrespondenzadresse

Dr. med. Hans-Jürgen Bartz, MBA
Leitung Geschäftsbereich Qualitätsmanagement
und klinisches Prozessmanagement
Universitätsklinikum Hamburg-Eppendorf
Martinistr. 52
20246 Hamburg
Deutschland
h.bartz@uke.de

Inhalt:
Aktueller Stand des klinischen Risikomanagements (kRM) in Deutschland im Jahr 2015 im Vergleich zu 2010. Übersicht der Studienergebnisse. Modifiziert nach Manser et al. [16]

Aktueller Stand des klinischen Risikomanagements (kRM) in Deutschland im Jahr 2015 im Vergleich zu 2010. Übersicht der Studienergebnisse. Modifiziert nach Manser et al. [16]. Die Skalierung wurde vereinfacht (siehe Spalte 3). Bewertung im Ampelformat: Grün = Verbesserung. Gelb = Status idem. Rot = Verschlechterung. Die Originalfragen können leicht anhand der Nummerierung in der Originalpublikation nachvollzogen werden.

Frage	Thema	Antwort	2010	2015	Bewertung
4.2.1 a	Zertifizierung	Ja	90%	73%	
4.2.1 b	Strategie kRM	Ja	27%	39%	
4.2.1 c	Strategische Ziele	Ja	18%	33%	
4.2.1 d	Operative Ziele	Ja	28%	32%	
4.2.1 e	Verantwortlichkeiten festgelegt	Ja	38%	60%	
4.2.1 f	Prozesse beschrieben	Ja	27%	60%	
4.2.1 g	Prozesse kommuniziert	Ja	40%	62%	
4.2.1 h	Fortbildungen	Ja	25%	67%	
4.2.1 i	Agenda der Klinikleitung	Ja	39%	88%	
4.2.1 j	Richtlinien umgesetzt	Ja	22%	77%	
4.2.1 k	Professionelle Beratung nötig	Ja	62%	67%	
4.2.1 l	Wo Beratung erhalten?	7 Themen	n. z.	n. z.	
4.2.1 m	Fallkonferenzen	Ja	73%	94%	
4.2.1 n	MMK	Systematisch	25%	59%	
4.2.2 a	Krankenaktenanalysen	Systematisch	14%	45%	
4.2.2 b	Beschwerdemanagement	Systematisch	75%	96%	
4.2.2 c	Schadensfälle auswerten	Systematisch	40%	64%	
4.2.2 d	Beobachtung klinischer Abläufe	Systematisch	29%	43%	
4.2.2 e	Kennzahlennutzung	Systematisch	50%	79%	
4.2.2 f	Befragungen Patienten/Angehörige	Systematisch	28%	91%	
4.2.2 g	Mitarbeiterbefragungen	Systematisch	38%	26%	
4.2.2 h	Hinweise Haftpflichtversicherer	Systematisch	30%	57%	
4.2.2 i	Dialogorientierte Verfahren	Systematisch	25%	28%	
4.2.2 j	Gesamtanalyse	Systematisch	19%	27%	
4.2.3 a	CIRS	Systematisch	35%	75%	
4.2.3 b	Einrichtungsübergreifendes CIRS	Systematisch	9%	22%	
4.2.3 c	Definition kritische Ereignisse	Ja	92%	96%	
4.2.3 d	Schadensfälle ins CIRS einbezogen	Ja	39%	35%	
4.2.3 e	CIRS anonym	Ja	97%	98%	
4.2.3 f	CIRS ermöglicht Rückfragen	Ja	78%	40%	
4.2.3 g	Schulungen CIRS	Immer	20%	60%	
4.2.3 h	Feedback an Meldende	Immer	36%	66%	
4.2.3 i	CIRS-Meldungen einsehen	Immer	35%	41%	
4.2.3 j	CIRS: Festes Schema zur Analyse	Immer	60%	83%	
4.2.3 k	CIRS: Maßnahmen abgeleitet	Immer	38%	89%	
4.2.3 l	CIRS: Maßnahmen kommuniziert	Immer	40%	68%	
4.2.3 m	CIRS: Maßnahmenumsetzung geprüft	Immer	42%	56%	
4.2.4 a	Patientenidentifikation	Systematisch	44%	76%	
4.2.4 b	Elektronische Patientenakte	Systematisch	20%	23%	
4.2.4 c	MRSA-Screening	Systematisch	72%	94%	
4.2.4 d	Sturzprävention und -assessment	Systematisch	65%	89%	

Frage	Thema	Antwort	2010	2015	Bewertung
4.2.4 e	Perioperative Checklisten	Systematisch	44%	87%	
4.2.4 f	Simulatortraining	Systematisch	14%	25%	
4.2.4 g	Entlassungsmanagement	Systematisch	45%	66%	
4.2.5 a	Statistik nosokomiale Infektionen	Systematisch	68%	93%	
4.2.5 b	Anzahl von Schadensfällen	Systematisch	34%	72%	
4.2.6 a	Ansprechperson für kRM	Trifft zu	37%	23%	
4.2.6 b	Regelmäßiger Austausch kRM und Nutzer	Trifft zu	44%	21%	
4.2.6 c	Vernetzung zwischen Abteilungen	Trifft zu	41%	17%	
4.2.6 d	Verantwortlichkeiten Führung festgelegt	Trifft zu	42%	21%	
4.2.6 e	Standardisierte klinische Prozesse	Trifft zu	35%	12%	
4.2.6 f	Offener Umgang mit Fehlern	Trifft zu	53%	23%	
4.2.6 g	Zusätzliche personelle Ressourcen	Trifft zu	38%	31%	
4.2.7 a	Förderung der Sicherheitskultur	Sehr relevant	44%	22%	
4.2.7 b	Risikoanalyse und -bewertung	Sehr relevant	35%	26%	
4.2.7 c	Risikoüberwachung	Sehr relevant	28%	21%	
4.2.7 d	Risikobeeinflussung und -steuerung	Sehr relevant	36%	21%	
4.2.7 e	CIRS	Sehr relevant	33%	14%	
4.2.7 f	Simulatortraining	Sehr relevant	11%	11%	
4.2.7 g	Kommunikation nach kritischem Ereignis	Sehr relevant	28%	21%	
4.2.9 a	Vergleich mit anderen Einrichtungen	> Durchschnitt	24%	36%	
4.2.9 b	Eigene Einschätzung zum Stand des kRM	In Prozent	39%	49%	
